# Characterization of Proteins Extracted from *Ulva* sp., *Padina* sp., and *Laurencia* sp. Macroalgae Using Green Technology: Effect of *In Vitro* Digestion on Antioxidant and ACE-I Inhibitory Activity

**DOI:** 10.1021/acsomega.3c05041

**Published:** 2023-12-14

**Authors:** Eda Şensu, Eda Nur Ayar, Emine Şükran Okudan, Beraat Özçelik, Aysun Yücetepe

**Affiliations:** † Department of Food Engineering, Faculty of Chemical and Metallurgical Engineering, Istanbul Technical University, Maslak TR-34469, Istanbul, Turkey; ‡ Department of Food Technology, Istanbul Gelisim Higher Vocational School, Istanbul Gelisim University, Avcılar, Istanbul 34310, Turkey; § Faculty of Fisheries, Akdeniz University, Dumlupınar, 07058 Antalya, Turkey; ∥ BIOACTIVE Research & Innovation Food Manufac. Indust. Trade Ltd., Katar Street, Teknokent ARI-3, B110, Sarıyer 34467, Istanbul, Turkey; ⊥ Department of Food Engineering, Faculty of Engineering, Aksaray University, TR-68100 Aksaray, Turkey

## Abstract

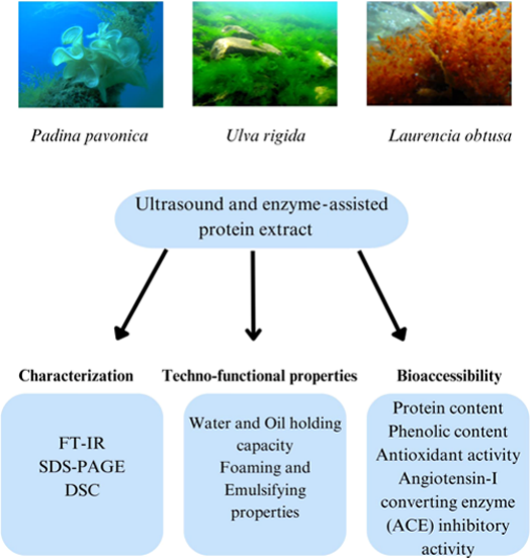

Macroalgal proteins were extracted from *Ulva rigida* (URPE) (green), *Padina pavonica* (PPPE) (brown), and *Laurencia obtusa* (LOPE) (red) using ultrasound-assisted enzymatic extraction, which is one of the green extraction technologies. Techno-functional, characteristic, and digestibility properties, and biological activities including antioxidant (AOA) and angiotensin-I converting enzyme (ACE-I) inhibitory activities were also investigated. According to the results, the extraction yield (EY) (94.74%) was detected in the extraction of *L. obtusa*, followed by *U. rigida* and *P. pavonica*. PPPE showed the highest ACE-I inhibitory activity before *in vitro* digestion. In contrast to PPPE, LOPE (20.90 ± 0.00%) and URPE (20.20 ± 0.00%) showed higher ACE-I inhibitory activity after *in vitro* digestion. The highest total phenolic content (TPC) (77.86 ± 1.00 mg GAE/g) was determined in LOPE. On the other hand, the highest AOA_CUPRAC_ (74.69 ± 1.78 mg TE/g) and AOA_ABTS_ (251.29 ± 5.0 mg TE/g) were detected in PPPE. After *in vitro* digestion, LOPE had the highest TPC (22.11 ± 2.18 mg GAE/g), AOA_CUPRAC_ (8.41 ± 0.06 mg TE/g), and AOA_ABTS_ (88.32 ± 0.65 mg TE/g) (*p* < 0.05). *In vitro* protein digestibility of three macroalgal protein extracts ranged from 84.35 ± 2.01% to 94.09 ± 0.00% (*p* < 0.05). Three macroalgae showed high oil holding capacity (OHC), especially PPPE (410.13 ± 16.37%) (*p* < 0.05), but they showed minimum foaming and emulsifying properties. The quality of the extracted macroalgal proteins was assessed using FTIR, SDS-PAGE, and DSC analyses. According to our findings, the method applied for macroalgal protein extraction could have a potential the promise of ultrasonication application as an environmentally friendly technology for food industry. Moreover, URPE, PPPE, and LOPE from sustainable sources may be attractive in terms of nourishment for people because of their digestibility, antioxidant properties, and ACE-I inhibitory activities.

## 1 Introduction

The world population is expected to reach 9.1 billion by 2050, a percent increase from now.^1^ Therefore, global agricultural production must be increased by 70% from the current levels to meet the food requirements of the higher population in 2050.^2^ Thereby, there is a growing development of emerging food technologies that promise to generate functional and bioactive ingredients, particularly protein-rich, for promoting human health.^3^ As protein demand increases with expanding populations, alternative protein sources are required for more environmentally friendly production. Compared to animal-based proteins, plant proteins appear to be the alternative to animal proteins due to their nutrient-rich composition (e.g., vitamins, minerals, fibers, proteins, and antioxidants) and their lower environmental effects, increasing sustainability.^4^ This has prompted scientists to look into new protein sources such as algae, legumes, fungi, and insects.^5^

Macroalgae are a valuable and sustainable protein source, from a nutritional standpoint. Macroalgae present faster growth, low water consumption (or even growth in seawater), higher photosynthesis efficiency, and carbon storage ability compared to plant-based protein sources.^4^ Macroalgae can be the ideal choice for meeting a sizable portion of the world’s food needs while having the least negative effects on the environment because they can absorb 10 to 50 times more CO_2_ than land plants.^6^ In addition, protein yield of macroalgae is provided 2.5–7.5 tons/ha/year per unit of land, while soybean and wheat yield are obtained as 0.6–1.2 tons/ha/year and 1.1 tons/ha/year, respectively. In this context, it should be stated that the world’s macroalgae production reached 23.4 million tons and an economic value of 6.4 billion in 2013. Moreover, it is known that 75% of the world’s freshwater resources should be used for protein production from plant and animal sources. Animal protein production requires 100 times more fresh water than is needed to produce an equivalent amount of vegetable protein. Macroalgae, on the other hand, do not require fresh water or arable land for their growth.^7^

The macroalgae have also gained widespread recognition for their significance as a source of functional components like minerals, polyphenols, carotenoids, proteins, and fibers due to their numerous health benefits. Hence, the isolation and research of novel components, such as proteins with biological activity derived from macroalgae, have received attention.^8^ Apart from their potential as a protein source, macroalgal proteins can also produce bioactive peptides and other proteinaceous compounds with biological value and positive effects on health. These beneficial effects include antioxidant, antiproliferative, anti-inflammatory, antihypertensive, antidiabetic, antiatherosclerotic, anticoagulant, and antimicrobial activity.^4^

Hypertension (high blood pressure) is an important risk factor for many cardiovascular diseases that affects approximately 20% of adult population worldwide.^9,10^ Blood pressure is regulated by different biochemical reactions. One of them is the renin-angiotensin system, which is one of the main components of blood pressure regulation physiology. Blood pressure is regulated by angiotensin-converting enzymes (ACE). Angiotensinogen produces angiotensin I, which ACE transforms into angiotensin II, which raises blood pressure. Angiotensin-converting enzyme activity is inhibited by the ACE inhibitor, which controls blood pressure.^11−14^ According to several publications, *in vitro* digestion causes considerable alterations in the proteins of various macroalgae that result in evidence of antioxidant and ACE-I inhibitory actions.^15−22^

*Padina pavonica* is a classified brown macroalgae that contributes significantly to the total productivity of marine environments.^23^ Red macroalga *Laurencia obtusa* produces secondary metabolites having medicinal properties,^24^ and *U. rigida* is a green macroalga that provides nutritional advantages while also assisting in the preservation of marine biodiversity. Furthermore, these species contain bioactive compounds with potential applications in the food industry, biomedicine, and cosmetics, demonstrating their ability to enhance both the environment and human health.^25^

These algae are rich sources of protein and potential candidates for use in human and animal nutrition. Studies have shown that *P. pavonica* contains 5.2–7.8% of dry weight (dw) protein,^26^ while *L. obtusa* contains 2.3–15.7% dw protein.^27,24^*U. rigida* has also been reported to contain high amounts of protein, up to 24% dw protein compared to terrestrial plants.^28,29^ The amino acid profiles of the proteins found in these macroalgae suggest that they are highly nutritious, with balanced amounts of essential amino acids.^30^ However, extracting these proteins is complex due to a strong cell wall.^7,31^ Improved extraction methods, such as cell disruption and specific chemical agents, can increase extraction efficiency while reducing drawbacks such as time, energy consumption, and protein integrity loss.^32,33^ Researchers have investigated the effect of combined ultrasound and enzyme on cell lysis.^34−37^

This study has applied a combination of pretreatments of osmotic shock and ultrasonication and a polysaccharidase enzyme to extract proteins from selected algae. In this context, the objectives of this study were to (i) perform protein extraction with high yield and characterize the protein extract of macroalgae, (ii) evaluate its techno-functional properties and the effect of *in vitro* gastrointestinal digestion on bioactivity, and (iii) comparison of protein extracts from *U. rigida*, *P. pavonica*, and *L. obtusa* in terms of bioactive, physicochemical, and techno-functional properties.

## 2 Materials and Methods

### 2.1 Materials

*U. rigida*, *P. pavonica, andL. obtusa* were collected from the Aegean coast of Türkiye (coordinates: 40°14′27.03″K 26°32′29.74″D, 40°14′27.03″K 26°32′29.74″D, 40°19′1.80″K 26°13′6.21″D, respectively). The collected algae were first washed with water to remove foreign materials such as epiphytes, rock, sand, and salt and then air-dried in a shaded place at ∼30 °C. The dried algae were ground into powder particles using a laboratory-type grinder (Waring 8011 Eb Blender, Cole-Parmer Instrument Company, Illinois) and sieved using a sieve (mesh size of 500 μm). The powdered macroalgae with <500 μm particle diameter was packaged appropriately to avoid sunlight and oxygen and stored at −20 °C until further analysis.

All of the solvents and chemicals used were of analytical or high-performance liquid chromatography grade. Hemicellulase enzyme (HSP 50000) was purchased from Bakezyme.

### 2.2 Protein Solubility and Surface Charge of Macroalgae

The protein solubility assay of the powdered macroalgae was evaluated as a function of pH (2–13) and carried out according to the method of Morr et al.^38^ Protein content in the supernatant was determined by the Lowry method^39^ The protein solubility (%) of the powdered macroalgae was calculated using eq 1
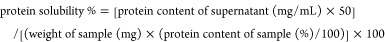
1

The net surface charge (zeta potential) was measured as a function of pH using a Nano-ZS instrument (Zetasizer NanoZS90, Malvern Instruments, U.K.).

### 2.3 Ultrasound and Enzyme-Assisted Extraction of Macroalgal Proteins

The combined ultrasound and enzyme-assisted extraction method was used to extract protein from three macroalgae.^40,41^ Briefly, 1 g of powdered macroalgae was mixed with 100 mL of citrate buffer solution (0.1 M, pH 4.5) and the mixture was kept at 4 °C overnight to induce cell lysis by osmotic shock. Then, the suspension was sonicated at a frequency of 53 kHz and 65% amplitude using an ultrasound homogenizer (Sonopuls HD 2200, Bandelin Electronic GmbH & Co. KG, Berlin, Germany). After the ultrasonication, hemicellulase enzyme was added to the suspension and kept in a shaking water bath (N-Biotek-303, Biotek Co., Ltd.) at different temperatures at 75 rpm for 24 h. Finally, the samples were kept in the shaking water bath at 85 °C for 10 min for enzyme inactivation. Then, the pH of the mixture was adjusted to the pH value where the protein solubility of macroalgae was the highest value determined based on the protein solubility assay, and the samples were again kept in the shaking water bath at 35 °C for a certain period for the second extraction. Then, the mixture was centrifuged at 18,782*g* (10,000 rpm in a Hettich 1720 Rotor, Hettich Rotina 380R, Germany) for 15 min. After centrifugation, the supernatant was collected and stored in the dark at −20 °C until analysis.

### 2.4 Determination of Protein Content and Extraction Yield

The modified Lowry method, which includes precipitating the proteins from the samples with trichloroacetic acid (TCA) to remove any potentially interfering compounds, was used to measure the protein content (PC).^42^ To determine extraction yield, the crude protein of the macroalgae was obtained by Association of Official Analytical Chemists methods.^43^ Bovine serum albumin was used as the standard protein. Protein content was expressed as milligrams of bovine serum albumin equivalents per gram of dry weight (mg of BSA/g of sample dw).

The extraction yield was calculated using eq 2
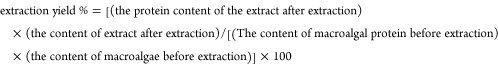
2

### 2.5 Total Phenolic Content

The total phenolic content (TPC) of the macroalgal protein extract was determined according to the Folin-Ciocalteu method.^44^ Gallic acid was used as standard, and the results are expressed as milligrams of gallic acid equivalents per gram of dry weight (mg of GAE/g dw).

### 2.6 *In Vitro* Biological Activities

#### 2.6.1 Angiotensin-I-Converting Enzyme (ACE-I) Inhibitory Activity

The *in vitro* ACE-I inhibitory activities of the macroalgal protein extracts were determined by the formation of hippuric acid. For the determination of ACE-I inhibitory activity, Martínez-Alvarez et al.’s^45^ method was revised and used. Briefly, 5 mM HHL, sample, and ACE (100 mU) were prepared in 100 mM sodium phosphate buffer (pH 8.3) containing 300 mM NaCl. Then, 200 μL of HHL and 50 μL of sample were mixed and incubated at 37 °C for 10 min. After 10 min, 20 μL of ACE enzyme was added to the mixture and incubated for 60 min at 37 °C in the shaking water bath. The enzymatic reaction was stopped by adding 250 μL of 1 M HCL. The released hippuric acid (HA) was then quantified by HPLC.

ACE-I inhibitory activity was quantified by an HPLC system (SPD M20A, Shimadzu) on an analytical C18 column (4.6 mm × 150 mm × 5 μm). The sample was separated by passing 0.8 mL/min with an injection volume of 10 μL. Water containing 0.1% (v/v) TFA (eluent A) and acetonitrile containing 0.1% (v/v) TFA (eluent B) were used as the mobile phases. A linear gradient flow of 20% B was passed through the column for 5 min and then 60% B for the next 15 min. The elution was held isocratically at 60% B for 4 min and then returned to the initial eluent composition of 20% B. Elution peaks of hippuric acid and HHL were detected at 228 nm.

ACE inhibition (%) was calculated as follows
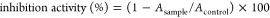
3where *A*_sample_ and *A*_control_ express the relative areas (*A*) of the HA peak of the assays performed with and without ACE inhibitors, respectively.

#### 2.6.2 Antioxidant Activity (AOA)

##### 2.6.2.1 Cupric Reducing Antioxidant Capacity Method

The cupric reducing antioxidant capacity (CUPRAC) assay was developed by Apak et al.^46^ Trolox was used as the standard, and the results are expressed as milligrams of Trolox equivalent per gram of dry weight (mg TE/g dw).

##### 2.6.2.2 2,2-Azinobis 3-Ethylbenzothiazoline-6-sulfonic Acid Diammonium Salt Method

2,2-Azinobis 3-ethylbenzothiazoline-6-sulfonic acid diammonium salt (ABTS) assay was performed according to Miller and Rice-Evans.^47^ Results were expressed as milligrams of TE/g dw.

### 2.7 Techno-Functional Properties

#### 2.7.1 Water and Oil Holding Capacity

The water holding capacity (WHC) and oil holding capacity (OHC) were evaluated according to the method of Kumar et al.^48^ The WHC/OHC of samples was expressed as the weight of water/oil absorbed per gram of the tested samples according to eq 4
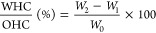
4where *W*_0_ is the weight of protein extract (g), *W*_1_ is the weight of the tube containing protein extract (g), and *W*_2_ is the weight of the tube after decantation of water and oil (g).

#### 2.7.2 Foaming Properties

The foaming properties were estimated using the method of Jarpa-Parra et al.^49^ The foaming capability (FC) and foaming stability (FS) were calculated using eqs 5 and 6
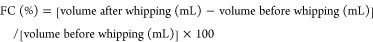
5
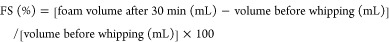
6

#### 2.7.3 Emulsifying Properties

The emulsifying activity (EA) and emulsion stability (ES) were evaluated with the method described by Tan et al.^50^ The emulsion activity of the samples was calculated using eq 7
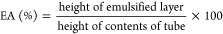
7

The ES of the samples was calculated using eq 8
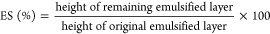
8

### 2.8 Characterization of the Protein Extracts

#### 2.8.1 Fourier Transform Infrared (FTIR) Spectroscopy

Organic groups in the macroalgal protein extracts were determined using FT-IR spectroscopy (Bruker Tensor II FTIR spectrometer equipped with the ATR diamond module (Bruker Optics, Germany)). All of the spectra were an average of 18 scans from 4000 to 400 cm^–1^ at a resolution of 4 cm^–1^.

#### 2.8.2 Sodium Dodecyl Sulfate-Polyacrylamide Gel Electrophoresis (SDS-PAGE)

SDS-PAGE experiments of macroalgal protein extracts were carried out on a Bio-Rad Mini-Protean Tetra Cell (Bio-Rad Laboratories, Inc., California). The separating gel (12% (w/v) acrylamide in 25 mM Tris–HCl (pH 8.9), 0.18 M glycine, and 0.1% SDS (sodium dodecyl sulfate)) and stacking gel (5% (w/v) acrylamide in 1.0 M Tris–HCl (pH 8.9), 0.18 M glycine, and 0.1% SDS) were prepared. The separation was performed at 110 V for approximately 90–60 min. Coomasie Brilliant Blue was used to dye the protein bands. The size markers (11–245 kDa) were purchased from an Opti-Protein XL Marker (Applied Biological Materials, Inc., Richmond, Canada).

#### 2.8.3 Differential Scanning Calorimetry (DSC)

The thermal properties of macroalgal protein extracts were determined by DSC (DSC 60 Plus, Shimadzu Instruments, Japan). Briefly, 20 mg of macroalgal protein extracts was placed in aluminum capsules. An empty aluminum capsule was taken as a reference. Run conditions were as follows: rate of heating, 10 °C/min; temperature range, 25–125 °C.

### 2.9 Simulated Gastrointestinal Digestion

*In vitro* gastrointestinal digestion of the macroalgal protein extracts was carried out according to the INFOGEST method.^51^ The collected samples taken after gastric and intestinal digestion were centrifuged at 10,000 rpm and 4 °C for 15 min and then stored at −80 °C until analysis.

### 2.10 Statistical Analysis

Statistical analysis was carried out using IBM SPSS Statistics 22 (Chicago) software. One-way ANOVA and the Tukey post hoc test were used to compare the treatments, and *p* < 0.05 was taken as a significant value. Microsoft Office Excel 2021 software (Microsoft Corporation) was used to calculate the correlation coefficients (*R*^2^).

## 3 Results and Discussion

### 3.1 Protein Solubility and Surface Charge

**Figure 1:**
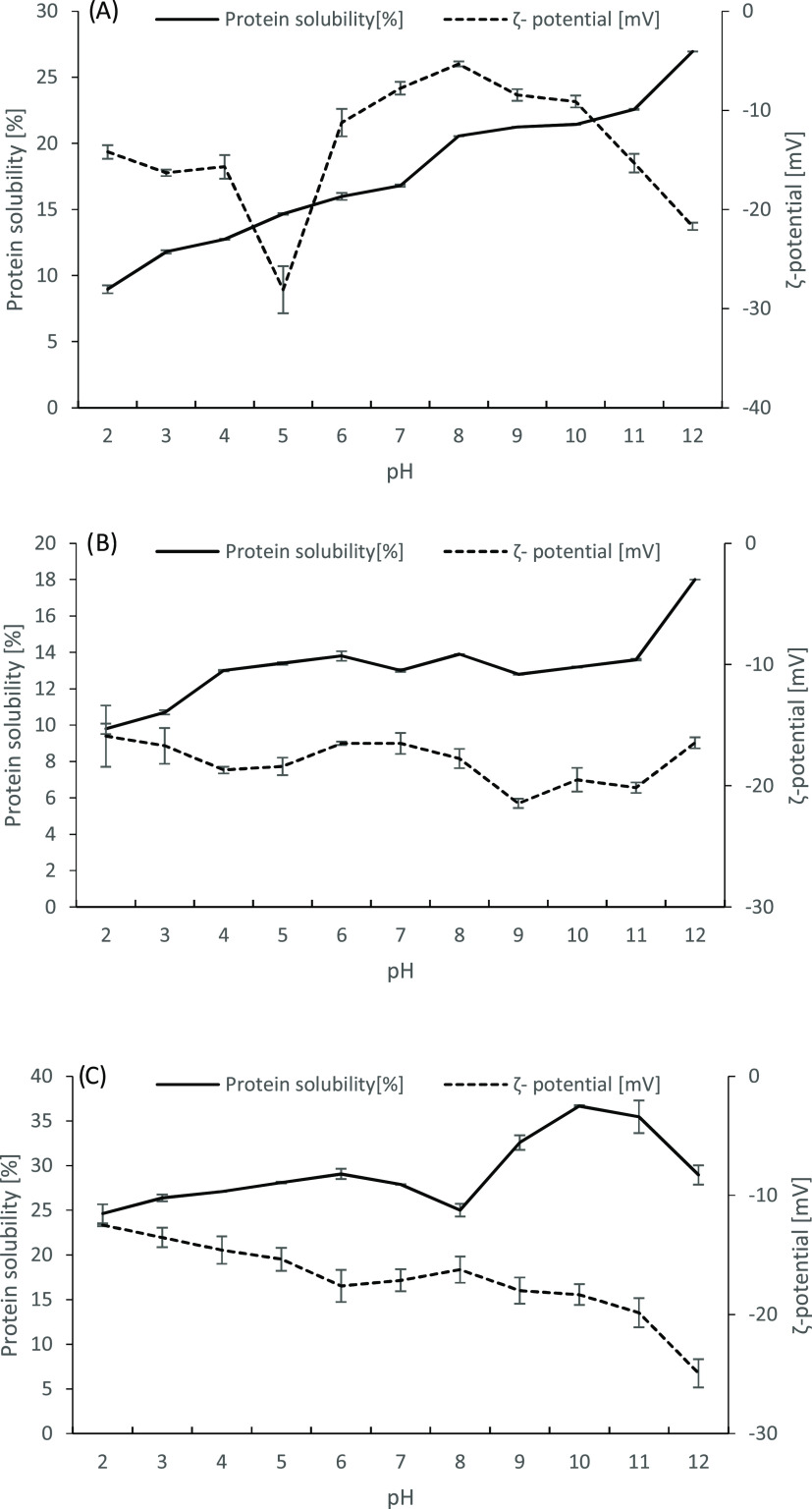
Effect of pH on net surface charge (ζ potential) and solubility of protein extract from (A) *U. rigida*, (B) *L. obtusa*, and (C) *P. pavonica*. Results are given as mean ± standard deviation.

The point at which macroalgae were most effectively dissolved before extraction was determined by analyzing the protein solubility. The net surface charges and protein solubility of the macroalgae were examined in relation to pH using zeta potential measurements (Figure 1). According to the results, *L. obtusa* and *P. pavonica* had the minimum surface charges at pH 2 (−15.9 ± 2.53 and −12.5 ± 0.09 mV, respectively), while the maximum surface charges were recorded above pH 9 (−21.46 ± 0.38 and −24.93 ± 1.18, respectively). However, the surface charges of *U. rigida* were the lowest at pH 8 (−5.33 ± 0.25 mV) and the highest at pH 5 (−28.1 ± 2.39). As seen in Figure 1, the ζ potential values of three macroalgae were found to be negative due to the presence of polysaccharides with negative charges in the extracts.^52,53^ Similarly, Shao et al.^53^ reported that the ζ potential of *Ulva fasciata* polysaccharides varied from −0.55 to −0.56 mV at pH values ranging from 5.0 to 10.0. Moreover, Wahlström et al.^54^ stated that the ζ potential of *Ulva* spp. varied between −53 mV and −59 mV at a neutral pH value and Rosenhahn et al.^55^ stated the ζ potential of *Ulva linza* as −19.3 mV at pH 8.2. Similar to these studies, Monsalve-Bustamante et al.^56^ obtained the ζ potential of *Gracilariopsis tenuifrons* as −31.0 at pH 7, as well. In seawater, the phosphate group is negatively charged. Other groups, some of which have positive charges, such as choline, may be present, but these positive groups will be overwhelmed by the negative charges, thus; the surface charge is affected by negative charges, which might be significantly more frequent.^55^

The surface charge and pH values of proteins have an important effect on their solubility. Three macroalgae had the minimum solubility at pH 2, which corresponded to their isoelectric point. On the other hand, *U. rigida* (36.44 ± 3.64%) and *L. obtusa* (19.8 ± 1.16%) showed the highest solubility at pH 13, while *P. pavonica* (36.7 ± 0.09%) had the highest solubility at pH 10. It has been shown that protein solubility increases at alkaline pH values and decreases under acidic conditions by some studies in the literature.^57,58^ For instance, Juul et al.^59^ reported that pH 2 was the isoelectric point of *Ulva spp.* and its solubility was the lowest level at this pH value. Harrysson et al.^60^ also obtained the lowest solubility for *Ulva lactula* at pH 2.0 as ∼12%, while the highest solubility was recorded at pH 12 as 62.1 ± 5.1%. Similarly, Bozdemir et al.^61^ reported that *Gracilaria dura* had maximum solubility (58.53 ± 4.26%) at pH 13. Likewise, Vilg and Undeland^58^ stated that the solubility of brown macroalgae *Saccharina latissima* showed the minimum value (30%) at pH 2–3 and the maximum value (100%) at pH 12. Algal proteins typically seem to have a lower isoelectric point than proteins from other biomasses, but it appears that only marine species do not exhibit an increase in solubility at a lower pH. This is due to an effect of the salt concentration used in the experiments; the well-known process for lowering the isoelectric point is the interaction of anions with positively charged protein groups at a low pH.^58,62^

### 3.2 Extraction Yield

The protein extraction yields for URPE, PPPE, and LOPE were determined to be 74.21, 63.20, and 94.74%, respectively (*p* < 0.05). The results of the present study are consistent with the literature. Fleurence et al.^63^ reported that the protein yield of *U. rigida* and *U. rodundata* doubled when the cellulase enzyme was used. Postma et al.^64^ observed a 25–30% increase in protein yield for *U. lactuca* using cellulase and pectinase enzymes. Mæhre et al.^40^ found that the protein yield of *Palmaria palmata* increased by approximately 1.6-fold when both cellulase and xylanase enzymes were used. Vásquez et al.^65^ investigated enzyme-assisted protein extraction from *Macrocystis pyrifera* and *Chondracanthus chamissoi* and found that the PC of the extract increased as a result of the breakdown of the cellulase-sensitive carbohydrate matrix. Harrysson et al.^60^ determined the extraction yield for *Ulva lactula* with traditional methods as 19.6 ± 0.8%. The extraction yield is largely influenced by the type of enzyme utilized and extraction conditions performed on the algae.^4^ For instance, Fleurence et al.^63^ studied the effect of polysaccharides on protein extraction using a combination of carrageenase and cellulase for *Chondrus crispus*, agarose, and cellulase for *Gracilaria verrucose*. They reported that these combinations increased the extraction yield 10-fold compared to that of untreated samples.

### 3.3 *In Vitro* Biological Activities

#### 3.3.1 Effect of *In Vitro* Digestion on Protein

In our previous study, the crude protein content of *L. obtusa* (red algea), *U. rigida* (green algae), and *P. pavonica* (brown algea) was found to be 116.5 ± 0.72 mg/g dw (11.65%), 74.15 ± 0.12 mg/g dw (7.41%), and 57.28 ± 0.12 mg/g dw (5.78%), respectively (*p* < 0.05).^66^ As observed in this study, it is known that the color of algae has an impact on their protein content.^67^ Red seaweeds are widely recognized to contain the highest protein content among macroalgae, whereas green macroalgae can have higher protein content than brown macroalgae.^68^ After ultrasound and enzyme-assisted protein extraction, PC of URPE, PPPE, and LOPE was determined to be 160.21 ± 0.29 mg BSA/g dw, 205.09 ± 0.54 mg BSA/g dw, and 227 ± 0.01 mg BSA/g dw, respectively (Table 1). According to Saravanavel & Pillai,^69^ the PC of macroalgae extracted by conventional methods was determined to be 15.08 mg/g dw for P. pavonica, 24.54 mg/g dw for *Ulva fasciata*, and 29.28 mg/g dw for L. obtusa. Compared with our results, the PC of these macroalgae extracted by traditional methods was found to be lower. Similarly, it is well established in the literature that ultrasound-assisted enzyme extraction has been shown to enhance protein content and extraction yield.^63,4^

**Table 1:** Changes in Protein Content of Macroalgal Protein Extract before and after *In Vitro* Digestion^a^

		*U. rigida*	*P. pavonica*	*L. obtusa*
PC (mg BSA/g)	before *in vitro* gastrointestinal digestion	160.21 ± 0.29^b,x^	205.09 ± 0.54^c,x^	227 ± 0.01^a,x^
	after *in vitro* gastric digestion	15.99 ± 0.61^b,z^	2.78 ± 0.61^c,z^	66.95 ± 6.0^a,y^
	after *in vitro* intestinal digestion	25.08 ± 3.21^a,y^	12.13 ± 0.01^b,y^	22.10 ± 1.60^a,z^

a Values are expressed as mean ± standard deviation for triplicate determinations. Different letters in the rows represent statistically significant differences (*p* < 0.05). Different superscript letters within the same line (a, b, c) and column (x, y, z) indicate significant difference (*p* < 0.05, Tukey).

The digestibility of macroalgal proteins in human gastrointestinal conditions is crucial for their utilization as human food.^70^*In vitro* digestion analysis was performed on URPE, PPPE, and LOPE, and the results are given in Table 1. During *in vitro* gastric phase, 90.02 ± 0.23% of URPE, 98.65 ± 0.18% of PPPE, and 70.50 ± 1.64% of LOPE were hydrolyzed. After *in vitro* intestinal phase, the percentages of protein hydrolyzed were 84.35 ± 2.01% for URPE, 94.09 ± 0.00% for PPPE, and 90.26 ± 0.70% for LOPE. Similarly, Kazir et al.^70^ reported that *Ulva sp.* and *Gracilaria sp.* proteins showed 47.8 ± 4.3 and 68.1 ± 0.7% digestion rates during *in vitro* gastric phase, respectively. Moreover, these proteins were highly digestible during the *in vitro* intestinal phase, with digestion rates of 89.4 ± 2.6 and 100% for *Ulva sp.* and *Gracilaria sp.* proteins, respectively. *In vivo* studies by Goni et al.^71^ have shown that various macroalgae contain a significant proportion of indigestible protein, ranging from 2% to 24%. Based on these results, it appears that the macroalgal protein extracts can undergo hydrolysis by digestive enzymes, potentially enhancing their absorption in the intestine.^70^

#### 3.3.2 Effect of *In Vitro* Dilution on Phenolics

In this study, a wide variation in the total phenolic content of the macroalgal protein extracts analyzed was obtained. The TPC for URPE, PPPE, and LOPE was found to be 19.32 ± 1.02 mg GAE/g dw, 49.92 ± 2.31 mg GAE/g dw, and 77.86 ± 1.0 mg GAE/g dw, respectively (Table 2). Red macroalgae *L. obtusa* has been found to possess the highest phenolic content compared to brown macroalgae *P. pavonica* and green macroalgae *U. rigida* (*p* < 0.05). Yuan et al.^72^ stated that the TPC of conventional extracts from some brown macroalgae was between 0.38 ± 0.01 and 0.78 ± 0.05 mg GAE/g, while the TPC of microwave-assisted extracts was 0.73 ± 0.02 and 1.4 ± 0.1 mg GAE/g. Wang et al.^73^ extracted TPC from *Palmaria palmata* using carbohydrase and protease enzymes and reported that TPC obtained by using the protease enzymes was found to be 3 times higher than the extract obtained without enzyme. In addition, the TPC of macroalgae might vary depending on location, environmental conditions, and seasonal fluctuations besides the novel extraction process, enzymes, and solvent.^74^

**Table 2:** Changes in Total Phenolic Content of Macroalgal Protein Extract before and after *In Vitro* Digestion^a^

		*U. rigida*	*P. pavonica*	*L. obtusa*
TPC (mg GAE/g)	before *in vitro* gastrointestinal digestion	19.32 ± 1.02^c,y^	49.92 ± 2.31^b,x^	77.86 ± 1.00^a,x^
	after *in vitro* gastric digestion	33.57 ± 1.92^b,x^	35.02 ± 2.13^b,y^	77.47 ± 1.78^a,x^
	after *in vitro* intestinal digestion	8.74 ± 1.34^b,z^	19.22 ± 0.09^a,z^	22.11 ± 2.18^a,y^

a Values are expressed as mean ± standard deviation for triplicate determinations. Different letters in the rows represent statistically significant differences (*p* < 0.05). Different superscript letters within the same line (a, b, c) and column (x, y, z) indicate significant difference (*p* < 0.05, Tukey).

To investigate the effect of digestion on TPC, *in vitro* gastric and intestinal digestion assays were performed on macroalgae. In the present study, TPC of *U. rigida*, *P. pavonica*, and *L. obtusa* was 33.57 ± 1.92 mg GAE/g, 35.02 ± 2.13 mg GAE/g, and 77.47 ± 1.78 mg GAE/g, respectively, after *in vitro* gastric digestion. On the other hand, after *in vitro* intestinal digestion, TPC values were reduced to 8.74 ± 1.34 mg GAE/g, 19.22 ± 0.09 mg GAE/g, and 22.11 ± 2.18 mg GAE/g, respectively (Table 2). At the end of the gastric phase, an increase in TPC can be observed in *U. rigida* and *L. obtusa*. A low pH value might encourage the release of phenols after breaking bonds within the matrix, including those of polysaccharides and proteins.^75^ At the end of the intestinal phase, TPC decreases due to the instability of phenols at high pH values.^76^ Similar to these findings, Corona et al.^77^ observed a significant reduction in TPC of brown macroalgae (*Ascophyllum nodosum*) after *in vitro* digestion, with a reduced level of 81.7%. In contrast, Huang et al.^78^ reported increased TPC after *in vitro* gastric digestion of seven macroalgae, especially for *Sargassum thunbergia* (174.44%). They also observed that the bound phenolic content of macroalgae remained relatively stable at 22.14–69.61% after *in vitro* intestinal digestion.^78^ These findings suggest that the *in vitro* digestion process has a variable impact on the TPC of macroalgae, which may be related to the species and type of polyphenols present. Furthermore, various factors can impact the absorption of phenolic compounds in the intestine, including pH, temperature, and food matrix.^79−81^

#### 3.3.3 Effect of *In Vitro* Digestion on the Antioxidant Activity

As seen in Table 3, AOA_CUPRAC_ of protein extracts obtained from URPE, PPPE, and LOPE was 22.40 ± 0.10 mg TE/g dw, 74.69 ± 1.78 mg TE/g dw, and 28.95 ± 2.31 mg TE/g dw, respectively (*p* < 0.05). On the other hand, AOA_ABTS_ of URPE, PPPE, and LOPE was 143.76 ± 3.2 mg TE/g dw, 251.29 ± 5.0 mg TE/g dw, and 187.34 ± 3.1 mg TE/g dw, respectively (*p* < 0.05) (Table 3). In our study, antioxidant activity assayed by CUPRAC and ABTS methods showed different trends since these methods have different mechanisms. They have different action modes in which CUPRAC allows the quantification of compounds capable of reducing the complex of Cu (II)-Neocuproine to Cu (I)-Neocuproine and ABTS allows the quantification of free radical scavenging capacity.^82,83^ On the other hand, CUPRAC and ABTS can test lipophilic and hydrophilic antioxidants simultaneously with the same precision due to the solubility of their single-charged chromophores in both aqueous and organic solvent environments.^84^

**Table 3:** Changes in Antioxidant Activity and Angiotensin-I-Converting Enzyme Inhibitory Activity of Macroalgal Protein Extract before and after *In Vitro* Digestion^a^

	AOA_ABTS_ (mg TE/g)			AOA_CUPRAC_ (mg TE/g)			ACE-I inhibitory activity (%)	
species	before *in vitro* gastrointestinal digestion	after *in vitro* gastric digestion	after *in vitro* intestinal digestion	before *in vitro* gastrointestinal digestion	after *in vitro* gastric digestion	after *in vitro* intestinal digestion	before *in vitro* gastrointestinal digestion	after *in vitro* intestinal digestion
*U. rigida*	143.76 ± 3.2^c,x^	60.24 ± 0.44^c,y^	58.42 ± 2.28^c,y^	22.40 ± 0.10^c,x^	2.90 ± 0.03^c,z^	6.72 ± 0.06^b,y^	2.90 ± 0.00^b^	20.20 ± 0.00^a^
*P. pavonica*	251.29 ± 5.0^a,x^	73.69 ± 0.17^b,y^	70.28 ± 2.91^b,y^	74.69 ± 1.78^a,x^	4.12 ± 0.25^b,z^	8.78 ± 0.00^a,y^	13.01 ± 0.00^a^	18.80 ± 0.00^b^
*L. obtusa*	187.34 ± 3.1^b,x^	85.23 ± 0.18^a,y^	88.32 ± 0.65^a,y^	28.95 ± 2.31^b,x^	6.87 ± 0.28^a,y^	8.41 ± 0.06^a,y^	0.30 ± 0.00^c^	20.90 ± 0.00^a^

a Values are expressed as mean ± standard deviation for triplicate determinations. Different letters in the rows represent statistically significant differences (*p* < 0.05). Different superscript letters within the same column (a, b, c) and line (x, y, z) indicate significant difference (*p* < 0.05, Tukey). AOA_ABTS_: Antioxidant activity by ABTS method, AOA_CUPRAC_: Antioxidant activity by CUPRAC method.

The protein extract from brown macroalgae *P. pavonica* has demonstrated higher antioxidant activity despite its low total phenolic content compared to the other samples. These findings suggest that coextracted bioactive compounds with antioxidant potencies, such as sulfated polysaccharides, tocopherols, proteins or peptides, and carotenoid pigments, may possess inherent antioxidant properties.^85^ Wang et al.^73^ investigated the AOA of *Palmaria palmata* extract obtained by using carbohydrase and protease enzymes and reported that enzyme-assisted extract indicated higher AOA than conventional extract. Yuan et al.^72^ reported that AOA_ABTS_ of microwave-assisted extracts from some brown macroalgae species was higher than that of conventional extracts, and the highest AOA found was 0.95 ± 0.01 mg TE/g.

The antioxidant activity of macroalgae is attributed to both amino acids with antioxidant properties and phenols. Besides, antioxidant activity and stability of macroalgal phenolic compounds are related to the type of algae, experimental temperature, and extraction conditions.^70,86^ In this work, after *in vitro* gastric digestion, *L. obtusa* showed the highest AOA_ABTS_ and AOA_CUPRAC_, followed by *U. rigida* and *P. pavonica* (*p* < 0.05). After *in vitro* intestinal digestion, AOA was found to be highest in *L. obtusa*, which contained the highest PC and TPC, followed by *P. pavonica* and *U. rigida* (*p* < 0.05). Similarly, Huang et al.^78^ stated that the AOA of six macroalgae markedly decreased after *in vitro* gastric digestion, while only the AOA of *Undaria pinnatifida* increased. Gonçalves et al.^87^ investigated the effect of digestion on the antioxidant activity of four wild edible plants and reported that antioxidant activity values significantly decreased after the gastric phase for all of the extracts and after the intestinal phase only for *P. major* extract. Additionally, after being digested *in vitro* system, proteins have been shown to have increased antioxidant activity in several investigations.^88,89^ According to Senphan and Benjakul,^88^ sea bass skin hydrolysate’s ABTS radical scavenging activity and chelating activity both slightly increased during pepsin digestion.

Hydrolysis can increase the antioxidant activity of proteins by releasing amino acid side groups that contribute to the antioxidant activity. The accessibility of amino acid residues inside the protein’s tertiary structure restricts its antioxidant activity prior to *in vitro* digestion. Antioxidant amino acids are exposed to more oxygen during the enzymatic hydrolysis process, which may increase their propensity to contribute hydrogen to the peroxyl radical.^90^ Moreover, antioxidant assay results may be impacted by the mode of action of antioxidants in various test systems and their localization in distinct food or biological system phases.^91^

#### 3.3.4 Effect of *In Vitro* Digestion on ACE-I Inhibitory Activity

The ACE-I inhibition activity of macroalgal protein extracts before and after *in vitro* digestion is demonstrated in Table 3. Among macroalgal protein extracts, PPPE had the highest ACE-I inhibitory activity (13.1 ± 0.00%) followed by URPE and LOPE (*p* < 0.05). To our knowledge, there have been few studies on the ACE-I inhibitory properties of macroalgae protein extracts. Cermeño et al.^92^ reported that the ACE-I inhibitory activity of *Porphyra dioica* protein extract was 14.57 ± 1.1%. Kumagai et al.^93^ stated that the *Pyropia pseudolineariz* protein inhibited ACE-I by 23.6%. Conversely, ACE-I inhibitory activity was found to be 79.87 ± 0.18% for *Ulva sp.* protein by Garcia-Vaquero et al.^94^ Based on these results, it can be concluded that the ACE-I inhibitory activity of macroalgal protein extracts from three different macroalgae is less than or comparable to that of other macroalgae. The difference in ACE-I inhibitory activity seen may be related to the primary structure of the protein, chain length, amino acid composition and sequences, and also extraction conditions.^95,96^

After *in vitro* digestion, the ACE-I inhibitory activity of macroalgae protein extracts increased. URPE (20.20 ± 0.00%) and LOPE (20.90 ± 0.00%) showed the highest ACE-I inhibitory activity after the partial hydrolyzed via pepsin and trypsin enzymes in the simulated gastrointestinal phase. According to Cermeno et al.,^92^ the ACE-I inhibitory activity of protein extract from *Porphyra dioica* after being hydrolyzed with alcalase and flavorzyme was determined to be 36.43 ± 3.4%. Garcia-Vaquero et al.^94^ reported that *Ulva sp*. protein hydrolyzed with papain inhibited ACE-I by 82.37 ± 0.05%. Hydrolysate of the *Pyropia pseudolineariz* protein showed 67.7% ACE-I inhibitory activity.^93^ Similarly, *Ulva intestinalis* (48.72 ± 1.13%) and *Gracilaria fisheri* (36.43–62.56%) protein hydrolysate showed the greatest ACE-I inhibitory activity after the hydrolyzation.^15,97^ Biparva et al.^98^ reported that the ACE-I inhibitory activity of *Macrocystis pyrifera* protein hydrolysate was 27.60 ± 0.005%. According to the literature, peptides are not active in the primary protein, but they can show their bioactive properties by being released by enzyme-catalyzed proteolysis *in vitro*.^20^ Similar to this, Pripp et al.^99^ reported that low-molecular-weight peptides exhibit stronger ACE-I inhibition activity compared to high-molecular-weight peptides.

### 3.4 Characterization Studies

#### 3.4.1 Techno-Functional Properties

##### 3.4.1.1 Water and Oil Holding Capacity

The water holding capacity (WHC) of the three macroalgal protein extracts is demonstrated in Table 4. It can be seen that URPE had the highest WHC (91.55 ± 0.11%) compared to PPPE (62.09 ± 5.49%) and LOPE (70.27 ± 0.01%) (*p* < 0.05). However, these results were lower than those reported for some algal proteins, such as *Enteromorpha compressa* (153 ± 0.07%), *E. tubulosa* (132 ± 0.11%), *E. linza* (122 ± 0.06%), *Kappaphycus alvarezii* (222 ± 0.04%), *Nannochloropsis oceanica* (287 ± 0.07%), *Chlorella pyrenoidosa* (202 ± 0.05%), *Arthospira platensis* (281 ± 0.04%), and *Gracilaria dura* (195 ± 0.08%).^61,100,101,48^ This might be a result of different polar amino acids influencing the protein–water interface and different extraction techniques.^100^ It is difficult to compare WHCs of different macroalgae samples with each other because of the wide range of chemical compositions, physical features, and extraction methods. Different protein conformations, the amount and character of water binding sites on protein molecules, and the types of water linked with the fibers all contributed to the chemical compositions. Additionally, physical parameters of samples, such as size and porosity, density, kinds of ions in solutions, and ionic strength, are important to fully comprehend the various behaviors of samples during hydration.^102−104^

**Table 4:** Techno-Functional Properties of Macroalgal Protein Extracts^a^

	WHC (%)	OHC (%)	EA (%)	ES (%)	FC (%)	FS (%)
*U. rigida*	91.55 ± 0.11^a^	397.47 ± 11.16^a^	33.26 ± 3.75^b^	20.46 ± 2.31^a^	28.92 ± 0.00^a^	11.56 ± 0.00^a^
*P. pavonica*	62.09 ± 5.49^b^	410.13 ± 16.37^a^	11.21 ± 0.00^c^	2.8 ± 0.00^c^	16.81 ± 2.5^b^	11.21 ± 2.5^a^
*L. obtusa*	70.27 ± 0.01^b^	182.32 ± 8.56^b^	46.33 ± 0.62^a^	7.17 ± 0.46^b^	5.61 ± 0.00^c^	0^b^

a Values are expressed as mean ± standard deviation for triplicate determinations. Different letters in the rows represent statistically significant differences (*p* < 0.05). WHC: Water holding capacity, OHC: Oil holding capacity, EA: Emulsifying activity, ES: Emulsion stability, FC: Foaming capacity, FS: Foaming stability.

The oil holding capacity (OHC) of three macroalgal protein extracts is shown in Table 4. PPPE (410.13 ± 16.37%) and URPE (397.47 ± 11.16%) had the higher OHC than LOPE (182.32 ± 8.56%) (*p* < 0.05). OHCs of three macroalgae were higher than those reported for some macroalgae, such as *K. alvarezii* (129 ± 0.20%), *E. compressa* (134 ± 0.10%), *E. tubulosa* (108 ± 0.04%), and *E. linza* (105 ± 0.07%) and some vegetable protein, such as soy protein isolate (360 ± 0.2%), whey protein isolate (190 ± 0.1%), and egg protein (210 ± 0.0%).^101,48,105^ In addition, The OHCs of whole *U. lactula* and *U. pertusa* were found to be 167 ± 0.59 and 153 ± 0.14%, respectively.^102,106^ In order to achieve the necessary functional properties in foods like meat, sausage, and mayonnaise, OHC is an important factor.^107^ Protein quantity, type, and amino acid composition impact OHC, especially the presence of hydrophobic groups in amino acids increases OHC.^108,67^ Further evidence that more hydrophobic proteins exhibit superior lipid binding suggests that nonpolar amino acid side chains bind the paraffin chains of fats, according to Kinsella’s^109^ investigation. As a result, proteins from three different macroalgae can be suitable candidates for the production of foods with improved lipid-binding capacity due to their high OHC.

##### 3.4.1.2 Foaming and Emulsifying Properties

The foaming capacity (FC) and foaming stability (FS) of three macroalgal protein extracts are shown in Table 4. FC of three different macroalgae varied between 28.92% (URPE) and 5.61% (LOPE). The FC of macroalgae was comparable to or higher than *E. linza* (15.6 ± 0.9%) while lower than *E. compressa* (40.9 ± 2.9%), *E. tubulosa* (45.0 ± 2.0%), and *K. alvarezii* (38 ± 2.0% at pH 6.0, 53.33 ± 2.31% at pH 4.0).^101,48^ In addition, FC of macroalgae was lower than some plant proteins such as soybean protein (65.7 ± 0.5%) and whey protein (132%).^110,111^ Ragab et al.^112^ emphasized that the solubility should be high for an effective foaming capability. Therefore, the FC of macroalgae can be explained as being related to their solubility profile of them. Moreover, Du et al.^113^ reported that lower FC could be caused by high levels of hydrophobic amino acids. Also, it has been reported that the increase in the surface charge of the proteins due to the pH change can make proteins more flexible and reduce hydrophobic interactions, thus enhancing foam formation.^114,115^ The low and negative net surface charge of macroalgae may also be a reason for its low foaming capacity. The FS can be influenced by several variables, including extraction procedure, macroalgal genotype, temperature, pH, and specific protein characteristics.^116,117^ URPE and PPPE exhibited ∼11.00% foaming stability in the present study, whereas LOPE showed no foaming stability. These values were higher than the FS of *E. linza* (4.4 ± 2.0%) but lower than the FS of *E. compressa* (37.5 ± 2.0%), *E. tubulosa* (16.7 ± 1.5%), and *K. alvarezii* (45.33 ± 1.15%).^91,47^

The emulsifying activity (EA) and emulsion stability (ES) of the three macroalgae are exhibited in Table 4. The highest EA was observed in LOPE (46.33 ± 0.62%) followed by URPE (33.26 ± 3.75%) and PPPE (11.21 ± 0.00%) (*p* < 0.05). URPE (20.46 ± 2.31%) has the highest ES compared to PPPE (2.8 ± 0.00%) and LOPE (7.17 ± 0.46%) (*p* < 0.05). Compared with other studies, EA and ES of *Gracilaria dura* were reported as 44 ± 0.00% and 75 ± 2.50%, respectively.^60^ Moreover, EA and ES of macroalgae were lower than some microalgal proteins such as *Chlorella vulgaris* (208.11 ± 0.22% for EA and 73.10 ± 4.68% for ES) and *Spirulina platensis* (51.54 ± 2.12% of EA and 65.20 ± 2.17% of ES).^118,119^ According to the literature, the proteins’ hydrophilic and hydrophobic qualities, net surface charge, and solubility may affect their EA and ES properties.^120,121^

#### 3.4.2 Structural Characterization

##### 3.4.2.1 FT-IR

**Figure 2:**
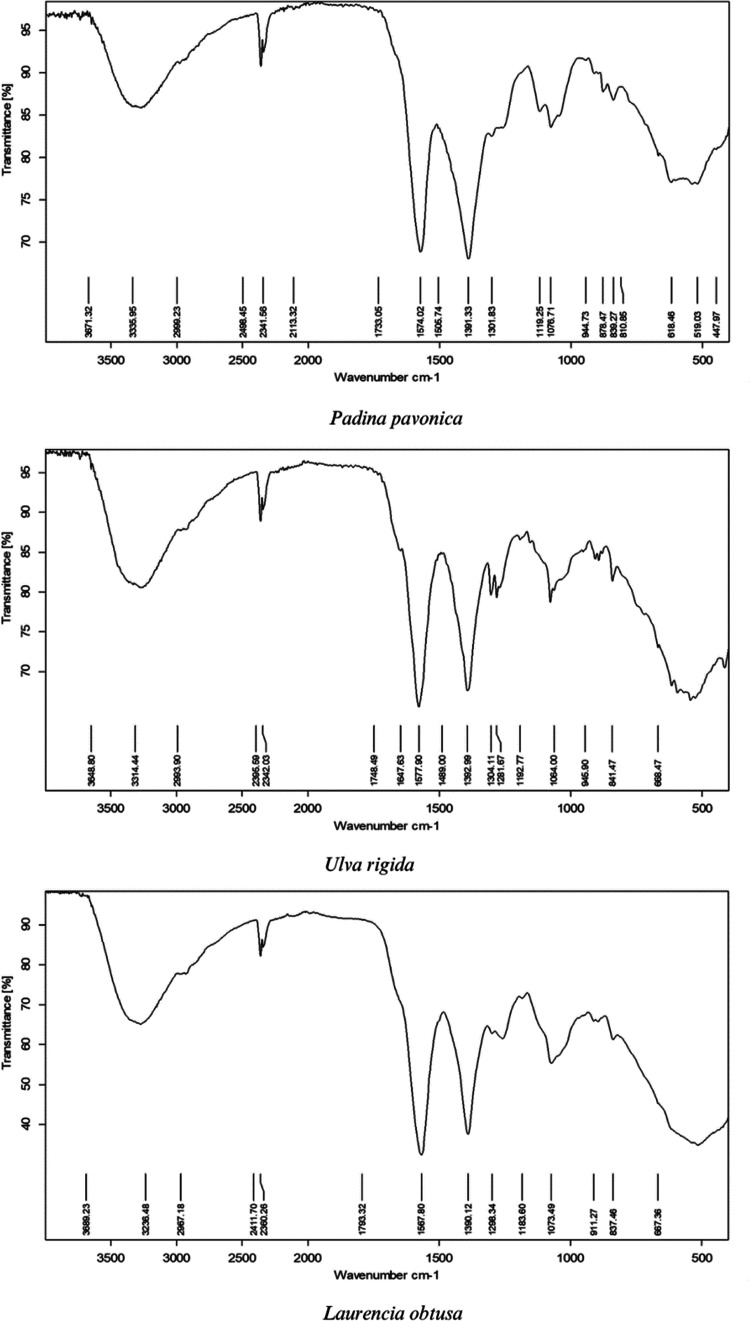
FTIR spectrum of *P. pavonica*, *U. Rigida*, and *L. obtusa* protein extract.

Proteins commonly contain a certain fraction of structural components such as α-helix, β-sheet, etc. In addition, determining protein secondary structures gives one of the most critical information for protein structure.^48^ Therefore, FT-IR spectrum has been used to estimate protein secondary structure (Figure 2). The FT-IR spectrum of the samples contained several typical bands for functional groups with variances in the absorption strength of some distinctive peaks. The broad band at ca. 3250–3350 cm^–1^ can be attributed to stretching vibrations of the O–H and N–H groups, stated Amide A.^122^ The peaks at ∼2900 cm^–1^ are attributed to the stretching vibrations of C–H groups, indicating the existence of neutral proteins, carbohydrates, and lipids.^65^ The Amide I band (∼1645 cm^–1^) results from C=O stretching, and the Amide II band (∼1530 cm^–1^) is due to the presence of stretching of C–N and bending vibrations of N–H groups.^113^ The existence of these two bands is indicative of the presence of proteinaceous in the samples.^123^ Similarly, Murdock and Wetzel^124^ indicated that cell walls of green algae contained abundant protein, implicating that strong amide I and amide II bands occurred at ∼1645 and 1530 cm^–1^, respectively. Moreover, peaks at the wavelength ranging ca. 1229–1301 cm^–1^ showed an amide III band stretching vibrations of C–N and N–H groups.^113^ The peaks ranging between 2400 and 2240 cm^–1^ for CO_2_ and peaks observed at 1850–1600 cm^–1^ correspond to C = O stretching vibration that may be caused by ketones, aldehydes, carboxylic acids, primary amides, and esters.^125^ In addition, the peaks at ca. 1000–1100 cm^–1^ are attributed to the C–O–C band, showing the existence of polysaccharides in macroalgal cell walls.^126^

##### 3.4.2.2 Sodium Dodecyl Sulfate-Polyacrylamide Gel Electrophoresis (SDS-PAGE)

**Figure 3:**
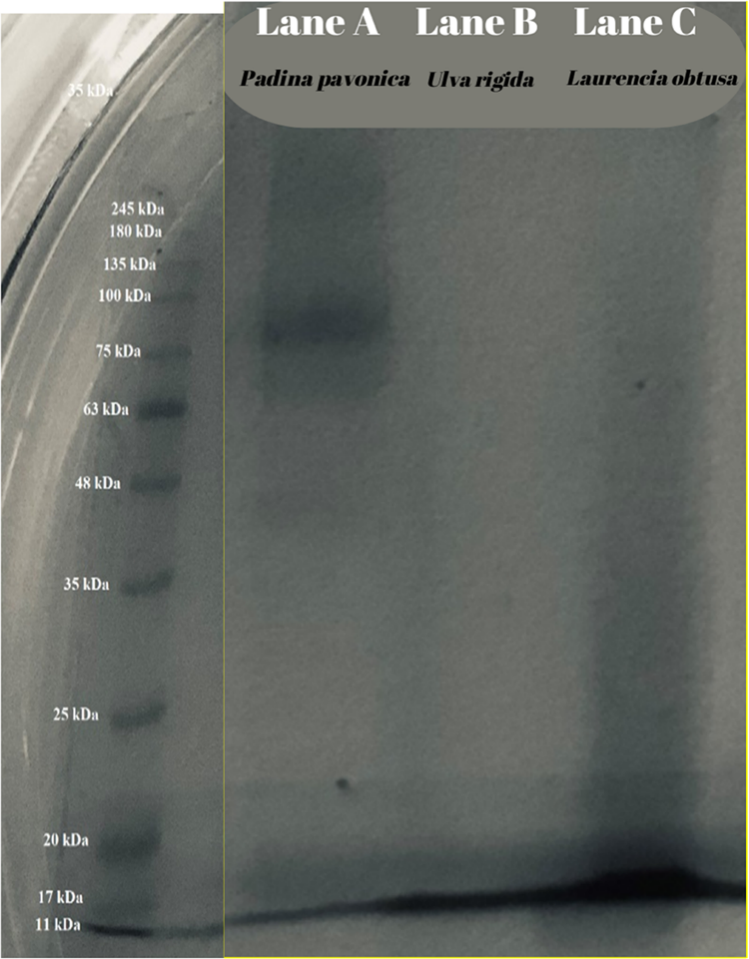
Protein bands by SDS-PAGE of *L. obtusa*, *U. Rigida*, and *P. pavonica* protein extract.

The SDS-PAGE patterns of extracted macroalgae proteins showed slightly different banding patterns among the different species in Figure 3. Comparable band patterns at low-molecular-weight peptides (<17 kDa) were observed in Lane A, Lane B, and Lane C of PPPE, URPE, and LOPE, respectively. URPE and LOPE displayed a similar protein profile with two protein bands observed at 11 and 17 kDa, with the exception of the more intense 11 kDa protein band in LOPE. PPPE had additional protein bands at 35, 48, and 75 kDa compared to URPE and LOPE. Consistent with our findings, Rouxel et al.^127^ also observed a limited number of protein bands in algae extract samples. Similarly, the electrophoresis pattern of *U. rigida* samples had low-molecular-weight bands (peptide bands <36 kDa, mainly peptides <12.3 kDa) close to those recorded in our study. In addition, the physical processing of algae might not alter the protein composition of algae.

##### 3.4.2.3 Differential Scanning Calorimetry (DSC)

**Figure 4:**
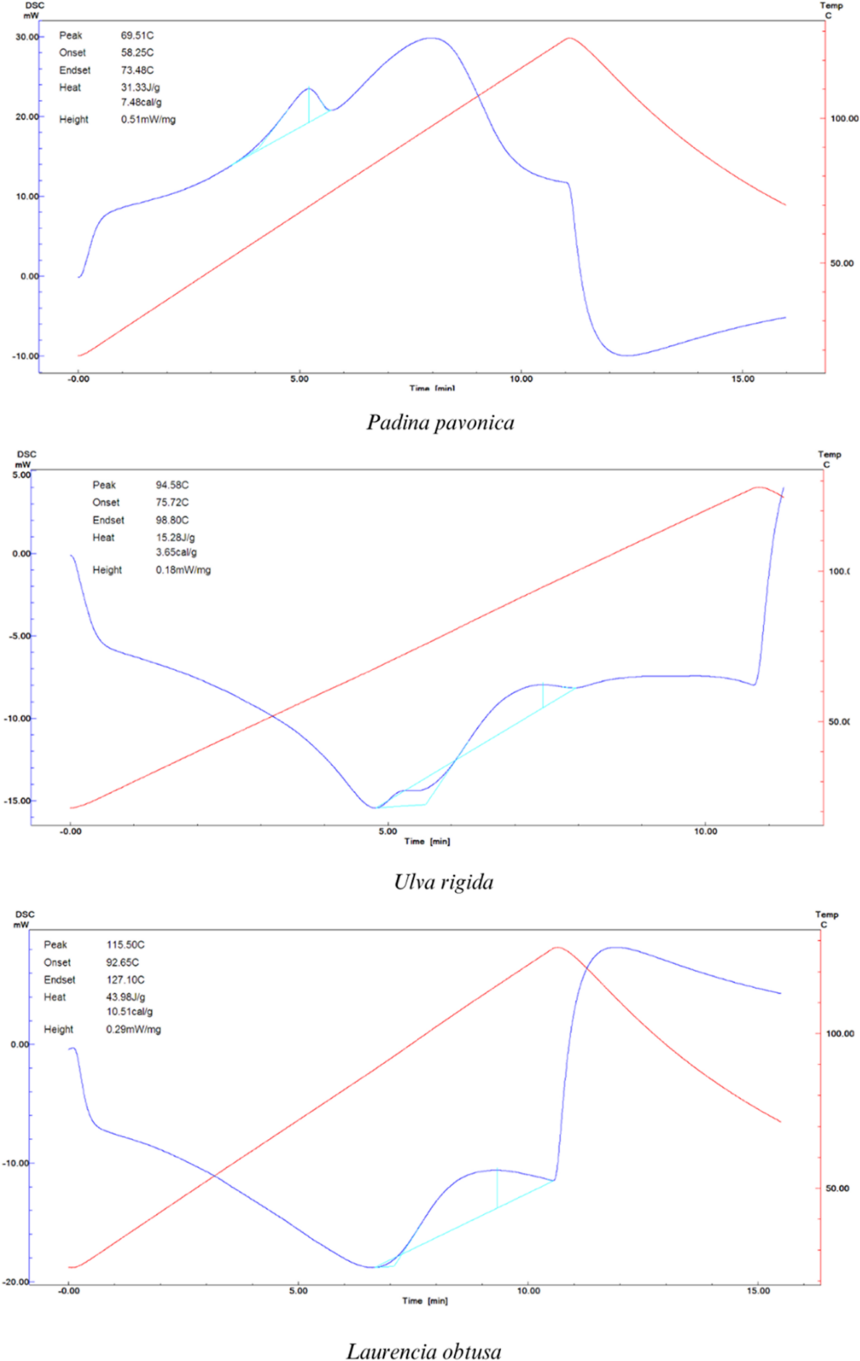
Thermal properties by DSC of *P. pavonica*, *U. Rigida*, and *L. obtusa* protein extract.

The thermal characteristics of macroalgal protein extracts are displayed in Figure 4. There were differences between the isolates in the denaturation enthalpy and temperature. The PPPE (69.51 °C) had the lowest denaturation temperature, while the URPE (94.58 °C) and LOPE (115.50 °C) showed noticeably greater denaturation temperatures. The denaturation enthalpy of PPPE, URPE, and LOPE was detected as 31.33, 15.28, and 43.98 J/g, respectively. The denaturation enthalpy measures the energy liberated during the reaction.^128^ All macroalgal proteins have different denaturation temperatures and enthalpies, which can be related to variations in the natural and chemical structures of the proteins. Rui et al.^129^ obtained protein from *Phaseolus vulgaris* legume varieties and reported that their denaturation temperature and enthalpy were ∼90 °C and ∼11 J/g. Gundogan and Karaca^128^ stated that various kinds of beans originating from Türkiye exhibited denaturation temperatures ranging from 90.5 to 152.4 °C, as well as corresponding denaturation enthalpies between 32.9 and 134 J/g. Compared with other vegetable proteins, the thermal stability of LOPE is similar to or higher than that of URPE and PPPE. The denaturation temperature and denaturation enthalpy can vary depending on the specific protein and the conditions of the experiment. A high denaturation temperature may indicate that the protein is heat-resistant. The homogeneity of the polypeptides, the type of bonding between the peptides, and the amino acid content of the protein are all factors that affect thermal stability.^130^ Moreover, the interactions between proteins and residual salts may increase heat stability in addition to changes in protein structure.^121^

## 4 Conclusions

In this study, we demonstrated that macroalgal protein could be considered an alternative source of protein, and we compared them to each other. Especially, *L. obtusa* had the highest protein content of 227 ± 0.01 mg BSA/g dw. The obtained macroalgal protein extracts have high antioxidant activity due to the presence of phenolic compounds. The utilization of macroalgae as a healthy food source for humans is supported by these antioxidant properties. Moreover, we developed/applied a novel extraction method including osmotic shock, enzyme, and ultrasound to improve the yield of macroalgal protein extraction. The highest extraction yield obtained for *L. obtusa* was 94.74%, following *U. rigida* (74.21%) and *P. pavonica* (63.20%). The three macroalgal protein extracts have similar functional properties to some commercial products in terms of water holding capacity, foaming capacity, stability, and emulsification activity, but they have a high oil holding capacity. Moreover, *L. obtusa* had higher thermal stability than *U. rigida* and *P. pavonica*. The structural conformation of macroalgal proteins had a significant impact on both their physicochemical and functional characteristics. In addition, during the *in vitro* intestinal phase, the digestibility of three macroalgal protein extracts was found to be remarkably high. The ACE-I inhibitory activity of LOPE and URPE was found to be 20.20 ± 0.00 and 20.90 ± 0.00% after *in vitro* gastrointestinal digestion, respectively. These results highlight the viability of employing macroalgae as a novel, renewable source of protein for human nutrition and commercial food processing. The antioxidant and ACE-I inhibiting peptides (compounds) in the macroalgal protein extract should be purified and identified in future pharmaceuticals or applications in food formulation.
