# Correction to “Characterization of Proteins Extracted from *Ulva* sp., *Padina* sp., and *Laurencia* sp. Macroalgae Using Green Technology: Effect of *In Vitro* Digestion on Antioxidant and ACE-I Inhibitory Activity”

**DOI:** 10.1021/acsomega.4c00407

**Published:** 2024-02-16

**Authors:** Eda Şensu, Eda Nur Ayar, Emine Şükran Okudan, Beraat Özçelik, Aysun Yücetepe

ACKNOWLEDGMENTS

This study was supported by The Scientific and Technological Research Council of Turkey (TUBITAK) (Project no: 119O149).

Details of corrections given above:

This manuscript is only an outcome of TUBITAK project (119O149). However, in the published manuscript (DOI: 10.1021/acsomega.3c05041), in addition to TUBITAK, Scientific Research Projects (BAP) Unit Research Fund of the Istanbul Technical University was also thanked by mistake.

